# Desmin aggrephagy in rat and human ischemic heart failure through PKCζ and GSK3β as upstream signaling pathways

**DOI:** 10.1038/s41420-021-00549-2

**Published:** 2021-06-26

**Authors:** Marion Bouvet, Emilie Dubois-Deruy, Annie Turkieh, Paul Mulder, Victoriane Peugnet, Maggy Chwastyniak, Olivia Beseme, Arthur Dechaumes, Philippe Amouyel, Vincent Richard, Nicolas Lamblin, Florence Pinet

**Affiliations:** 1 INSERM, Univ. Lille, CHU Lille, Institut Pasteur de Lille, U1167 - RID-AGE - Facteurs de risque et déterminants moléculaires des maladies liées au vieillissement, F-59000 Lille, France; 2 Normandie Univ, UNIROUEN, Inserm U1096, FHU-REMOD-VHF, 76000 Rouen, France

**Keywords:** Heart failure, Immunochemistry

## Abstract

Post-translational modifications of cardiac proteins could participate to left contractile dysfunction resulting in heart failure. Using a rat model of ischemic heart failure, we showed an accumulation of phosphorylated desmin leading to toxic aggregates in cardiomyocytes, but the cellular mechanisms are unknown. The same rat model was used to decipher the kinases involved in desmin phosphorylation and the proteolytic systems present in rat and human failing hearts. We used primary cultures of neonate rat cardiomyocytes for testing specific inhibitors of kinases and for characterizing the autophagic processes able to clear desmin aggregates. We found a significant increase of active PKCζ, no modulation of ubitiquitin-proteasome system, a defect in macroautophagy, and an activation of chaperone-mediated autophagy in heart failure rats. We validated in vitro that PKCζ inhibition induced a significant decrease of GSK3β and of soluble desmin. In vitro activation of ubiquitination of proteins and of chaperone-mediated autophagy is able to decrease soluble and insoluble forms of desmin in cardiomyocytes. These data demonstrate a novel signaling pathway implicating activation of PKCζ in desmin phosphorylation associated with a defect of proteolytic systems in ischemic heart failure, leading to desmin aggrephagy. Our in vitro data demonstrated that ubiquitination of proteins and chaperone-mediated autophagy are required for eliminating desmin aggregates with the contribution of its chaperone protein, α-crystallin Β-chain. Modulation of the kinases involved under pathological conditions may help preserving desmin intermediate filaments structure and thus protect the structural integrity of contractile apparatus of cardiomyocytes by limiting desmin aggregates formation.

## Introduction

Left ventricular (LV) remodeling following myocardial infarction (MI) is one of the main causes of cardiac dysfunction leading to heart failure (HF) [1, 2]. To date, several mechanisms implicated in LV remodeling such as infarct size, fibrosis, and apoptosis as well as alterations in contractile proteins and in intracellular calcium handling have been identified, however, the exact mechanisms underlying HF remain imperfectly known.

Using an experimental rat model of ischemic HF and phosphoproteomic technologies, we previously identified increased levels of serine phosphorylated desmin in the LV of ischemic HF rats [3, 4]. Desmin is a 53kDa muscle-specific intermediate filament protein, which forms a three-dimensional scaffold around the myofibrillar Z-disk and interconnects the contractile apparatus to the cellular organelles [5]. Desmin is the target of post-translational modifications (PTM), such as phosphorylation as well as nonenzymatic modifications such as glycation [6–8]. The major effect of phosphorylation is the disassembly of desmin filaments [6] that impacts the solubilization of insoluble intermediate filaments [9]. Glycogen synthase kinase 3 (GSK3) β has been described to be involved in desmin phosphorylation [10], which was shown recently to induce calpain-1-mediated muscle desmin depolymerization [11].

We previously showed the presence of most of the phosphorylated desmin in the insoluble LV protein fraction of ischemic HF rats [4] suggesting that desmin phosphorylation, could promote the formation of desmin aggregates in failing heart, as already observed in a canine pacing model of desynchronous HF [10, 12] as well as in desminopathies. In the latter case, the majority of desmin causative mutations lead to cardiac conduction abnormalities and to the accumulation of insoluble desmin-containing aggregates [13, 14]. Desmin phosphorylation cause disturbance of the cytoskeletal network, thus leading to loss of function of desmin linked to cardiomyocyte death and development of cardiomyopathy [15]. Interestingly, it has been previously shown that desmin expression and its pattern of striation correlated with the level of myocardial injury in patients with idiopathic dilated cardiomyopathy [16]. Thus, desmin and more particularly its phosphorylation and aggregation may contribute to cardiac toxicity and account for heart dysfunction during LV remodeling following MI [17]. The aggregates of hyperphosphorylated desmin present in HF hearts are proteotoxic [18] and may be due to inefficient autophagy processes [19].

The aim of the present study was therefore to decipher the kinases involved in the phosphorylation of desmin and to investigate the contribution of the different proteolytic systems such as the ubiquitin-proteasome system (UPS), macroautophagy, and chaperone-mediated autophagy (CMA) [20] in the accumulation of hyperphosphorylated desmin aggregates in human and experimental HF.

## Results

### Phosphorylated desmin is associated with active PKCζ and GSK3β in LV of HF-rats 2 months after MI

**Fig. 1:**
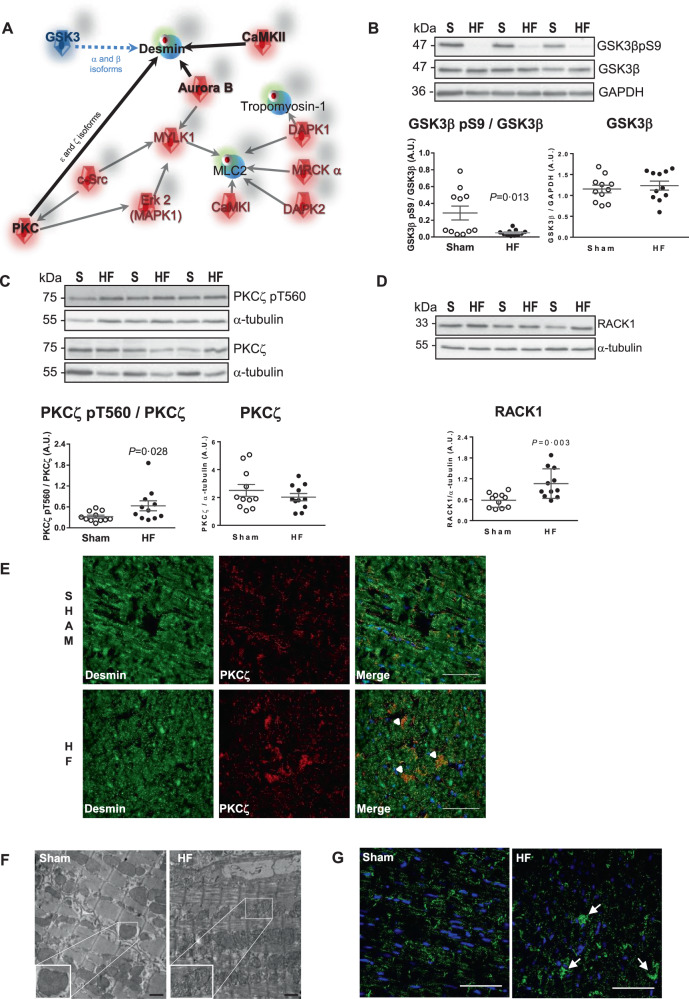
Desmin is phosphorylated by active PKC*ζ* and GSK3β in the LV of HF-rats 2 months after MI. **A** In silico screening of kinases potentially involved in desmin phosphorylation with GeneGo pathways software selected CaMKII, Aurora B, and PKC (ε and ζ isoforms). GSK3 was previously described to be involved in desmin phosphorylation [10]. **B** Representative western blots (top panel) and quantification of inactive GSK3β (GSK3β pS9/GSK3β ratio) (bottom left panel) and GSK3β levels (bottom right panel) in the LV of sham- (*n* = 11) and HF-rats 2 months post-MI (*n* = 11). **C** Representative western blots (top panel) and quantification of active PKCζ (PKCζ pT560/ PKCζ ratio) (bottom left panel) and PKCζ levels (bottom panel) in the same samples. **D** Representative western blot (top panel) and quantification of RACK1 levels (right panel) in the same samples. The loading controls (GAPDH and α-tubulin) are indicated on the graphs. Graphs show individual and mean ± SEM values expressed in arbitrary units (A.U.). Significant *P* values are indicated on the graphs. **E** Double immunofluorescence staining for desmin (green) with PKCζ (red) in the LV of sham- and HF-rats 2 months post-MI. Arrows indicate the colocalisation of desmin with PKCζ. Scale bar represents 60 µm. **F** LV ultrastructure of sham- and 7 days post-MI rats (top panel) and sham- and HF-rats 2 months post-MI (bottom panel). Higher magnifications of mitochondria from LV of 2 months sham- and post-MI rats are shown as insert. Scale bar represents 1 µm. **G** Immunofluorescence staining for desmin aggregates indicated by arrows only in LV of HF-rats compared to sham-rats with nuclei stained in blue. Scale bar represents 60 µm.

Phosphorylation is a dynamic process resulting from changes in phosphatases or kinases activities. As previously shown, neither the activity nor the protein levels of PP2A, a central cardiac phosphatase involved in the dephosphorylation of cardiac proteins [21] was significantly modulated in the LV of HF-rats [22]. Consequently, we selected four kinases by in silico screening (Fig. 1A), potentially involved in desmin phosphorylation, calmodulin-dependent protein kinase II (CaMKII), Aurora B, PKC (ε and ζ isoforms), and GSK3 (α and β isoforms). We investigated their expression in our rat HF model and excluded CaMKII and Aurora B. Indeed, active CaMKII (CaMKII pT286/CaMKII ratio) was completely decreased in the LV of HF-rats (Fig. S1A), in accordance with the decreased levels of phosphorylated phospholamban (PLB) (Fig. S1B). Aurora B was not significantly modulated in the LV of HF-rats (Fig. S1C) as confirmed by the phosphorylation levels of desmin at serine 60 [23] not modulated between the 2 groups of rats (Fig. S1C). This was validated by the nuclear localization of Aurora B without colocalization with desmin (Fig. S1C). We also excluded PKCε since its expression was decreased in the LV of HF-rats [22]. Then, we first evaluated GSK3 levels, recently shown to be involved in desmin phosphorylation in an experimental canine model of dyssynchronous HF [10], especially GSK3β for which its role in cardiac biology is well recognized [24]. GSK3β phosphorylation levels at S9 were significantly decreased in LV of HF-rats without any modulation of GSK3β levels, corresponding to a decrease of the inactive form (Fig. 1B). We also showed a significant decrease of the inactive form of GSK3α phosphorylated at S21 without any modulation of GSK3α levels (Fig. S1D). Finally, we showed an increase of PKCζ phosphorylated on T560 without any modulation of PKCζ levels (Fig. 1C) in the LV of HF-rats. These results suggest an increase of PKCζ active form in the LV of HF-rats validated by the increase of one of its scaffold protein, Receptor for Activated C Kinase 1 (RACK1) levels (Fig. 1D). Immunofluorescence staining showed a weak colocalization of desmin with PKCζ (Fig. 1E and Table S3) and with its anchoring protein RACK1 in HF-rats (Fig. S1E and Table S3). We observed by transmission electron microscopy (TEM) a stronger alteration of LV structure with sarcomeres disruption and mitochondrial structural abnormalities characterized by a loss of electron-dense matrix at 2 months post-MI (Fig. 1F). Desmin was mainly expressed in the insoluble fraction, suggesting the formation of hyperphosphorylated desmin aggregates, as highlighted by immunofluorescence staining in LV of 2 months post-MI rats (Fig. 1G). Interestingly, the levels of one of its chaperone protein, α-crystallin Β-chain (CryAB), were unchanged in both fractions (Fig. S1F), suggesting that desmin aggregates are not the results of CryAB defect. These results suggest that the presence of desmin aggregates may participate to myofilament architecture disruption and local cardiomyocyte function alteration as recently suggested [25].

### Dysregulated autophagy contributes to phosphorylated desmin aggregates in LV of heart failure rats

**Fig. 2:**
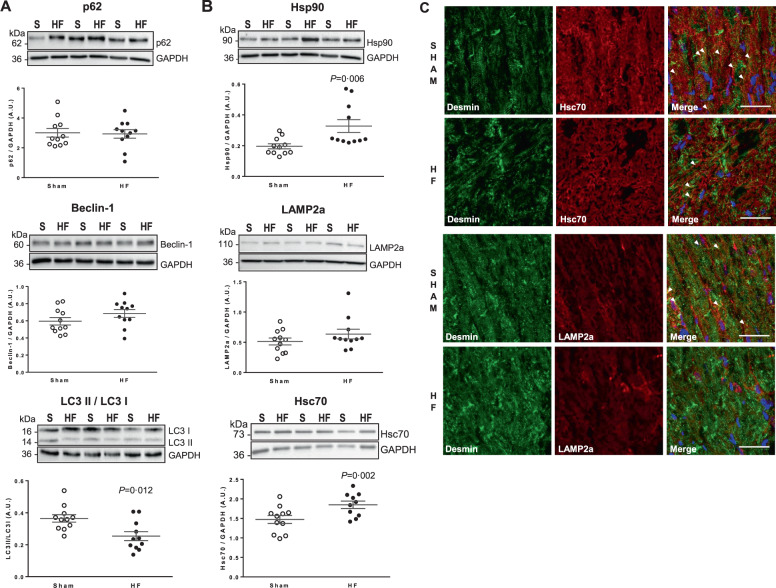
LV autophagy response to HF following MI. **A** Representative western blots and quantification of macroautophagy markers, p62 (top panel), beclin-1 (middle panel), and LC3 II/LC3 I ratio (bottom panel) in LV of sham- (*n* = 11) and HF-rats 2 months post-MI (*n* = 11). **B** CMA markers: Hsp90 (top panel), LAMP2a (middle panel), and Hsc70 (bottom panel) were quantified by western blots in the same samples. The loading controls (GAPDH) are indicated on the graphs. Graphs show individual and mean ± SEM values expressed in arbitrary units (A.U.). Significant *P* values are indicated on the graphs. **C** Double immunofluorescence staining for desmin (green) with Hsc70 (red) (top panels) and for desmin (green) with LAMP2a (red) (bottom panels) in LV of sham- and HF-rats 2 months post-MI. Nuclei are stained in blue. Arrows indicate the colocalization of desmin with respectively Hsc70 and LAMP2a. Scale bar represents 30 µm.

We explored the different proteolytic pathways (e.g., macroautophagy, CMA, and ubiquitin proteasome) in order to decipher their contribution in phosphorylated desmin clearance in our experimental model of HF. We showed an alteration of macroautophagy by a significant decrease of the LC3II/LC3I ratio with no modulation of p62, beclin-1, and LC3II levels in the LV of HF-rats (Fig. 2A and Fig. S2A). These data are correlated with the absence of double-membrane vesicles (Fig. 1F). We did not observe any modulation of ubiquitinated protein levels (Fig. S2B), TRIM32 [26] (Fig. S2C), and ASB2β [8] (Fig. S2D) between sham- and HF-rats. Interestingly, we found 4 KFERQ-like CMA motifs [27] in desmin sequence, conserved between species, suggesting that desmin may be a new CMA substrate. We found a significant increase of Hsp90 and Hsc70 levels but no modulation of LAMP2a levels in the LV of HF-rats (Fig. 2B). Altogether, our data show an impaired macroautophagy process in LV at 2 months post-MI and activation of CMA during the development of HF. The presence of KFERQ-like CMA motifs suggests that CMA may be involved in desmin clearance in the heart. This hypothesis was supported in part by a weak colocalization of desmin with LAMP2a (Fig. 2C).

Based on these new in vivo data about the potential implication of PKCζ on desmin phosphorylation and the modulation of macroautophagy and CMA on clearance of soluble and insoluble forms of desmin, we investigated these mechanisms in primary culture of rat neonatal cardiomyocytes (NCM).

### PKCζ modulate desmin phosphorylation in in vitro culture of cardiomyocytes

**Fig. 3:**
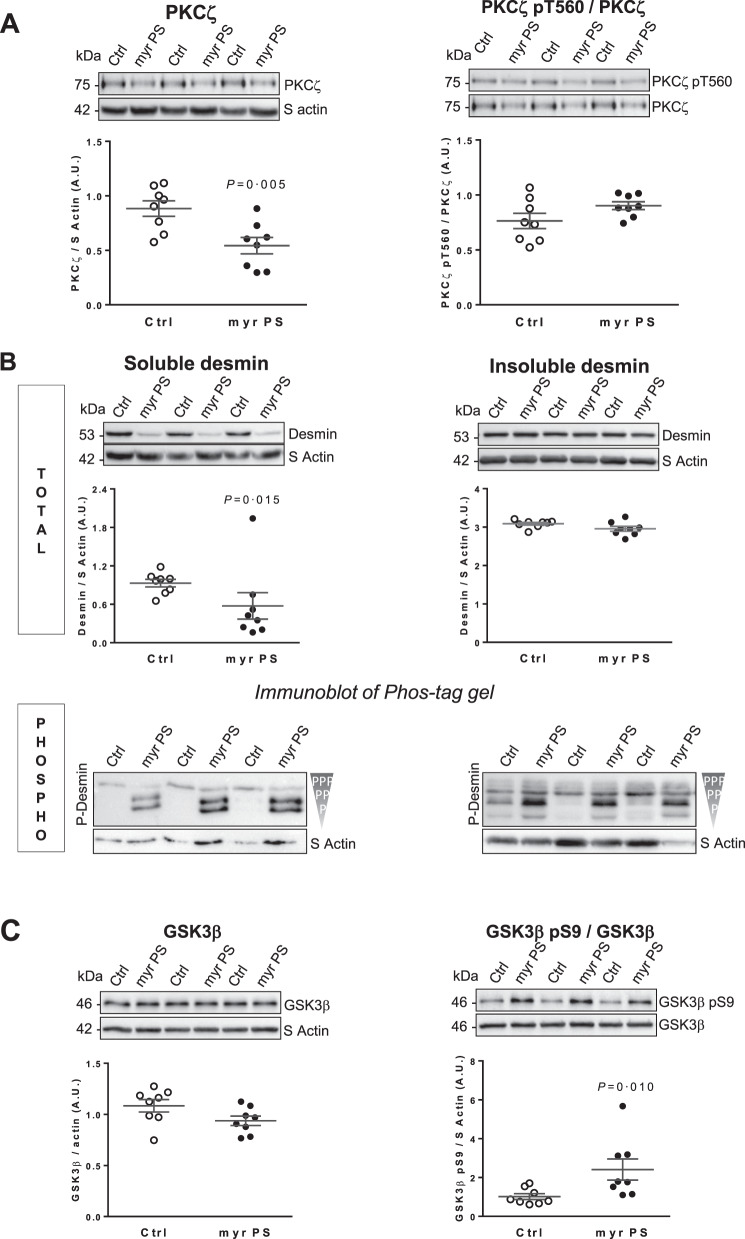
PKCζ modulates desmin phosphorylation in vitro in cardiomyocytes. **A** Representative western blots and quantification of PKCζ levels (left panel) and active PKCζ (PKCζ pT560/PKCζ ratio) (right panel) in control and NCM treated with 10 µmol/L of PKCζ inhibitor, myrPS for 1 h (*n* = 8). **B** Soluble (top left panel) and insoluble (top right panel) desmin levels and their phosphorylation profiles (Phos-tag gel, bottom panels) were analyzed in response to PKCζ inhibition in the same samples. Each band of the desmin immunoblot of Phos-tag gels represents a phosphorylated form of desmin, the upper band being the most phosphorylated and the lower band the less or no phosphorylated form of desmin. **C** Representative western blots and quantification of GSK3β levels (left panel) and inactive GSK3β (GSK3β pS9/GSK3β ratio) (right panel) in the same samples. The loading control (S Actin) is indicated on the graphs. Graphs show individual and mean ± SEM values expressed in arbitrary units (A.U.). Significant *P* values are indicated on the graphs.

For inhibiting specifically PKCζ, we used a cell-penetrating myristoylated (myrPS) peptide (^114^-IYRRGARRWRKL-^125^) [28]. Treatment of NCM by myrPS induced significant PKCζ inhibition with a decrease of PKCζ levels without any modulation of PKCζ pT560/PKCζ ratio (Fig. 3A). We observed a significant decrease of soluble desmin levels while insoluble desmin levels were unchanged upon PKCζ inhibition (Fig. 3B). Both soluble and insoluble (but to a lower extent) desmin phosphospecies, visualized by Phos-tag gels, showed less phosphorylated desmin in myrPS-treated NCM, suggesting less phosphorylation of desmin upon PKCζ inhibition (Fig. 3B, Fig. S3). Interestingly, we also observed a modulation of GSK3β upon PKCζ inhibition with a significant increase of the inactive form of GSK3β, leading to increased inactive GSK3β (Fig. 3C).

Interestingly, GSK3β inhibition with 6-Bromoindirubin-3’-oxime inhibitor (BIO, 2 and 5 µM), for which we verified specific inhibition of GSK3β by the significant modulation of GSK3β pS9/GSK3β ratio (Fig. S4A) without any modulation of GSK3β and of active PKCξ (PKCξ pT560/PKCξ ratio and PKCξ) (Fig. S4B), did not modulate soluble desmin levels (Fig. S4C, left and top panel) but induced only significant increase of insoluble desmin levels (Fig. S4C, right and top panel) for 2 µM of BIO. The phosphospecies distribution of soluble desmin was modulated with upper molecular weight desmin species upon GSK3β inhibition in contrast with the phosphospecies distribution of insoluble desmin which was not modulated (Fig. S4C, bottom panels). Another attractive feature is that the soluble and insoluble desmin phosphorylation profiles were different depending on whether PKCξ or GSK3β was inhibited (Fig. S4C, bottom panels) suggesting that the two kinases may directly phosphorylate desmin at different serine residues.

Altogether, our in vitro results suggest that PKCζ may regulate indirectly GSK3β as recently suggested [29] and that both kinases are involved on desmin phosphorylation in cardiomyocytes.

### Impact of macroautophagy and CMA modulation on desmin clearance in cardiomyocytes

Macroautophagy was induced by nutrient starvation with HBBS treatment with or without a 2 h pretreatment with bafilomycin to inhibit macroautophagic flux and thus increase its efficiency (Fig. S5). Under HBSS treatment, we found efficient macroautophagy activation quantified by a significant increase of LC3II and LC3II/LC3I ratio which was significantly increased with bafilomycin pretreatment (Fig. S5A). Soluble desmin levels were significantly degraded by nutrient starvation-induced autophagy (Fig. S5B). Interestingly, insoluble desmin levels were not modulated by HBSS treatment (Fig. S5C). These results confirmed the efficiency of macroautophagy in the degradation of soluble desmin in cardiomyocytes.

**Fig. 4:**
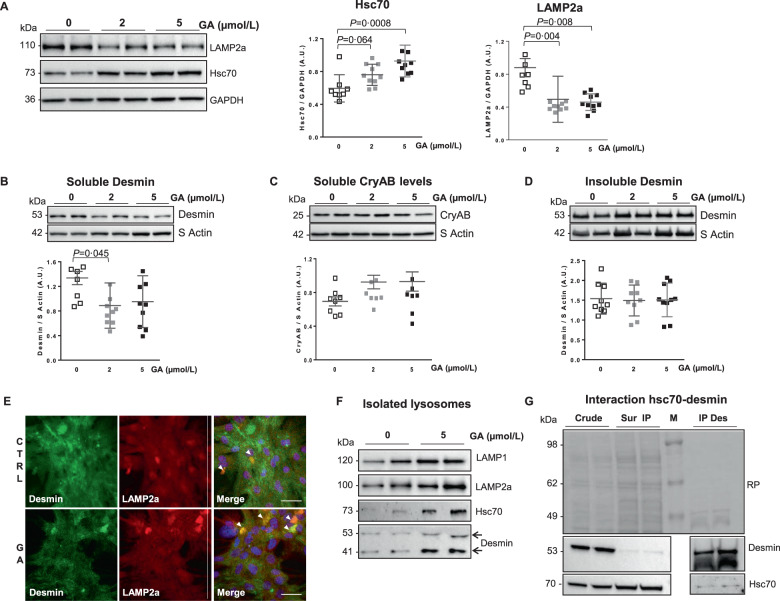
Clearance of soluble desmin by inducing CMA in vitro in cardiomyocytes. **A** Representative western blots and quantification of CMA markers: Hsc70 (left panel) and LAMP2a (right panel) in control (*n* = 8) and NCM treated with 2 and 5 µmol/L of geldanamycin (GA) during 17 h (*n* = 10). **B**, **C** The impact of CMA induction was evaluated by western blot on soluble protein fractions in the same samples for desmin (**B**) and CryAB (**C**). **D** The impact of CMA induction on insoluble desmin was evaluated by western blot in the same samples. The loading controls (GAPDH and S Actin) are indicated on the graphs. Graphs show mean ± SEM values expressed in arbitrary units (A.U.). Significant *P* values are indicated on the graphs. **E** Representative immunofluorescence staining of desmin (green) and LAMP2a (red) in control and NCM treated with 5 µmol/L of GA during 17 h. Nuclei are stained in blue. Arrows show the colocalization points. Scale bar represents 30 µm. **F** Representative western blots of lysosome marker (LAMP1), CMA markers (LAMP2a and Hsc70), and desmin in isolated lysosomes of H9c2 cells treated or not with 5 µmol/L of GA during 17 h. Arrows show the different desmin entities. **G** Detection of interaction between desmin and Hsc70 in H9c2 cells by immunoprecipitation of desmin followed by western blot for desmin and Hsc70. Crude: crude cells sample; Sur IP: supernatant of desmin immunoprecipitation. IP Des: immunoprecipitation of desmin; Efficiency of Des IP was verified with desmin western blot; M: size markers are indicated on the left side of red ponceau (RP) staining membrane.

To decipher the contribution of CMA in desmin clearance in cardiomyocytes, CMA was induced by either 2 or 5 µmol/L of geldanamycin (GA) for 17 h (Fig. 4 and Fig. S6A–B) as previously described [30]. Under GA treatment, we found a significant increase of Hsc70 (Fig. 4A), with a significant decrease of LAMP2a and soluble desmin levels (Fig. 4B) and no effect on its chaperone, CryAB (Fig. 4C). Interestingly, insoluble desmin levels were not modulated by GA treatment (Fig. 4D). Immunofluorescence staining showed a weak colocalization of desmin with LAMP2a in CMA-activated NCM (Fig. 4E), despite the significant decrease of LAMP2a in the whole-cell extract (Fig. 4A) that could be explained by its restricted lysosomal localization. To strengthen the demonstration that desmin is a CMA substrate, we isolated lysosomes from H9c2 cells treated or not with GA. In isolated lysosomes, verified by LAMP1 expression, we showed, as expected, an increase of LAMP2a and Hsc70 in isolated lysosomes upon GA treatment (Fig. 4F). Interestingly, the classical 53 kDa form and a cleaved form of 39 kDa of desmin were found in the lysosomes, which were strongly increased upon CMA activation (Fig. 4F). The cleaved form may be due to calpain proteolytic cleavage as previously described [31]. We also verified that desmin can interact with Hsc70 by immunoprecipitation of cardiac cells (Fig. 4G), showing the ability of Hsc70 to target desmin to the lysosomes.

**Fig. 5:**
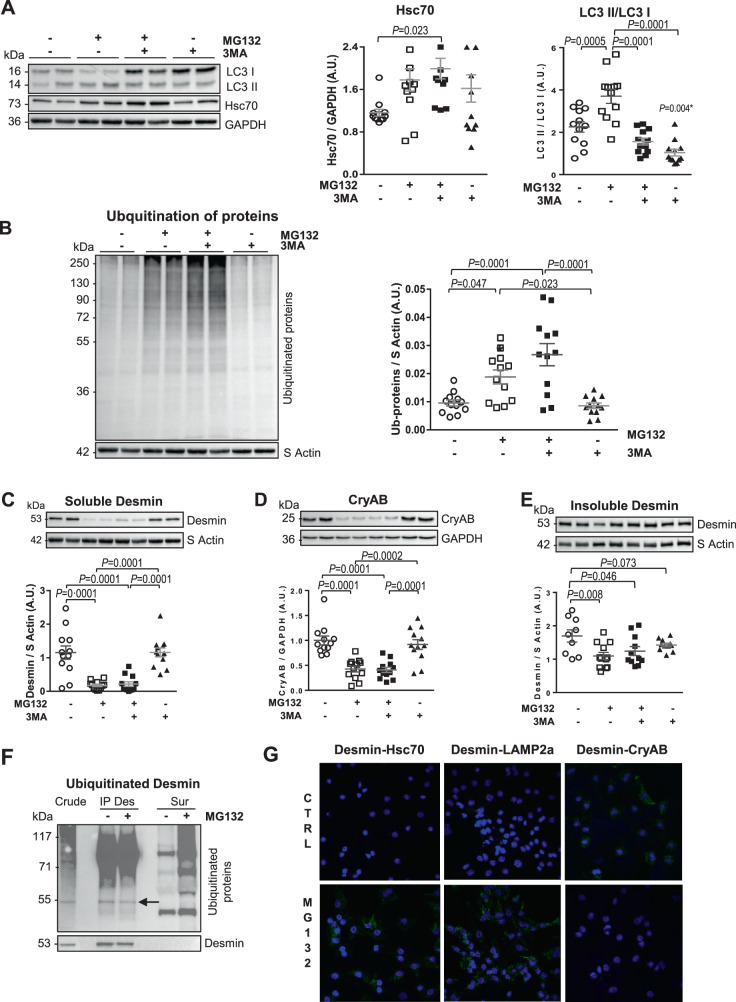
Clearance of soluble desmin in vitro in cardiomyocytes co-treated with 3MA and MG132. **A** Representative western blots and quantification of Hsc70 levels and LC3 II/LC3 I ratio in untreated/control cells (*n* = 12), NCM treated either with MG132 (10 µmol/L) (*n* =12 ) or 3-MA (10 mmol/L) (*n* = 12) for 18 h, and NCM pre-treated with 3-MA (10 mmol/L) for 1 h and then co-treated with 3-MA (10 mmol/L) and MG132 (10 µmol/L) for 18 h (*n* = 12). **B** Representative western blots and quantification of ubiquitylated protein levels in the same samples. **C** Representative western blots and quantification of soluble desmin levels in the same samples. **D** Representative western blots and quantification of soluble CryAB levels in the same samples. **E** Representative western blots and quantification of insoluble desmin levels in the same samples. The loading controls (GAPDH and S Actin) are indicated on the graphs. Graphs show individual and mean ± SEM values expressed in arbitrary units (A.U.). Significant *P* values are indicated on the graphs. *in panel **A** shows the significance of 3MA treatment *vs* untreated cells. **F** Detection of ubiquitinated desmin in control and NCM treated with MG132 (10 µmol/L) for 18 h by immunoprecipitation of desmin followed by western blot for ubiquitin and desmin. Crude: crude LV sample; IP Des: immunoprecipitation of desmin; Sur IP: supernatant of desmin immunoprecipitation. Efficiency of Des IP was verified with desmin western blot. **G** Representative PLA immunofluorescence staining of desmin-Hsc70, desmin-LAMP2a, and desmin-CryAB in control and NCM treated with MG132 (10 µmol/L) for 18 h. Nuclei are stained in blue.

We also checked the specificity of CMA activation in cardiomyocytes by analyzing the impact of GA on other protein degradation pathways. We found no modulation of the ubiquitin-proteasome pathway (Fig. S6A), but a significant decrease of macroautophagy markers in cardiomyocyte (Fig. S6B). Then, we used a combined pharmacological treatment with MG132, a proteasome inhibitor, and 3-methyladenine (3MA), a macroautophagy inhibitor. We verified that the co-treatment induced specifically CMA activation by the significantly increased Hsc70 levels as previously shown (Fig. 5A) and significant inhibition of macroautophagy assessed by LC3II/LC3I ratio (Fig. 5A), and LC3II, with no modulation of Beclin-1 and p62 levels (Fig. S6C). As expected, the inhibition of proteasome induced significantly increased levels of ubiquitinated proteins exacerbated upon inhibition of macroautophagy (Fig. 5B). With this other pharmacological treatment, we observed a significant decrease of soluble desmin levels induced by ubiquitination and CMA activation (Fig. 5C). Interestingly, CryAB expression was also significantly decreased (Fig. 5D) as well insoluble desmin levels (Fig. 5E). These data showed the role of desmin co-chaperone, CryAB for the clearance of insoluble desmin in cardiomyocyte. We observed low levels of ubiquitinated desmin, independently of proteasome inhibition, validated by the increased levels of ubiquitinated proteins in NCM treated by MG132 and the specificity of desmin immunoprecipitation by western blot (Fig. 5F). Immunofluorescence stainings using PLA suggested that desmin is a CMA substrate with a significantly increased colocalization of desmin with Hsc70 and LAMP2a and a significantly decreased colocalization of desmin with CryAB in NCM treated with MG132 (Fig. 5G). Despite a lack of modification of desmin ubiquitination levels found in the in vivo model, it is well known that proteasome impairment in the context of cardiac stress may induce aberrant protein aggregation in the heart nutrient starvation.

### Dysregulated autophagy associated with the levels of desmin and kinases in human heart failure patients

**Fig. 6:**
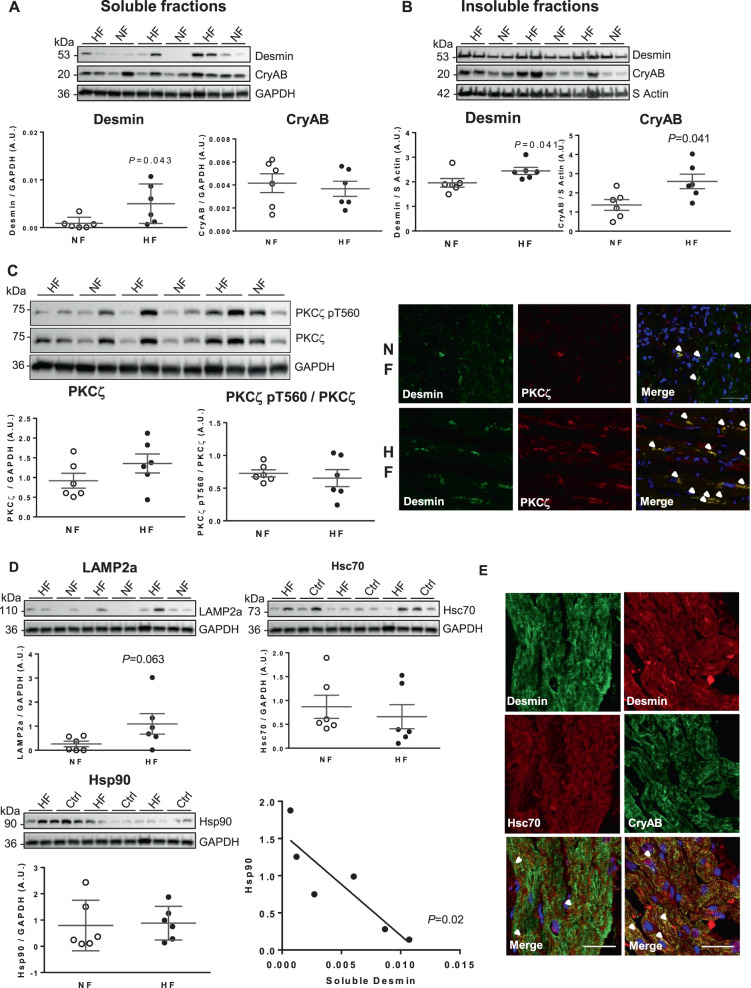
Desmin presents a scaffold defect and accumulates in the failing human hearts. **A**, **B** Representative western blots and quantification of soluble (**A**) and insoluble (**B**) desmin (left panels) and CryAB (right panels) levels in the heart of non-failing (NF) (*n* = 6) and failing (HF) patients (*n* = 6). **C** Representative western blots (top left panel) and quantification of PKCζ (bottom left panel) and active PKCζ (PKCζ pT560/PKCζ ratio) (bottom right panel) in the same samples. Double immunofluorescence staining for desmin (green) and PKCζ (red) (top panels) in frozen myocardial sections from non-failing (NF) and failing (HF) patients. Scale bar represents 20 µm. Nuclei are stained in blue as shown in the merge staining. Arrows indicate colocalization of desmin with PKCζ. **D** Representative western blots and quantification of LAMP2a (left panel), Hsc70 (middle panel), and Hsp90 (right panel) in the hearts of non-failing (NF) (*n* = 6) and failing (HF) patients (*n* = 6). Correlation analysis of Hsp90 expression levels with soluble desmin expression levels in the heart of failing patients (*n* = 6) is shown (bottom panel). The loading controls (GAPDH and S Actin) are indicated on the graphs. Graphs show individual and mean ± SEM values expressed in arbitrary units (A.U.). Significant *P* values are indicated on the graphs. **E** Double immunofluorescence staining for desmin (green) with Hsc70 (red) (left panels) and for desmin (red) with CryAB (green) (right panels) in frozen myocardial sections from failing patients. Scale bar represents 50 µm. Nuclei are stained in blue as shown in the merge staining. Arrows indicate colocalization of desmin with Hsc70 or CryAB.

Our studies based on the in vivo experimental rat model and the in vitro cardiomyocyte model prompted us to assess levels of desmin, as well as the kinases selected to be involved in desmin phosphorylation and the chaperone proteins such as CryAB and CMA markers in the failing hearts of patients (Fig. 6 and Fig. S7). Ubiquitination and macroautophagy markers were not modulated as previously described [32]. We found significantly increased desmin levels with no modulation of CryAB levels in soluble protein fractions in the failing hearts compared to non-failing human hearts (Fig. 6A). On the other hand, both insoluble desmin and CryAB levels were significantly increased in the failing hearts (Fig. 6B). Surprisingly, we showed no significant modulation of PKCζ and of active PKCζ (Fig. 6C), of inactive GSK3β (Fig. S5A) and, of active CaMKII (Fig. S5E) between the two groups of patients. As in the experimental rat model of HF, we observed a significant strong colocalization of desmin with PKCζ in failing human hearts compared to the non-failing hearts (Fig. 6C and Table S3). Among CMA markers, despite the low number of human samples analyzed and the inter-individual heterogeneity, LAMP2a levels tended to be increased in failing hearts while the levels of Hsc70 and Hsp90 were unchanged (Fig. 6D). Interestingly, Hsp90 and soluble desmin levels significantly correlate negatively in the failing hearts (Fig. 6D). We also observed a weak colocalization of desmin with Hsc70 and with its chaperone CryAB in failing and in non-failing human hearts (Fig. 6E, Fig. S7C, and Table S3). As also observed in rat model there is only a weak colocalization between desmin and LAMP2a in human hearts (Fig. S7D). Our data showed that the increase of insoluble desmin and CryAB, corresponding to desmin aggregates might be explained by alteration of both proteasome and autophagy activities.

## Discussion

The present study aimed to describe a novel signaling pathway implicating PKCζ as an upstream actor of GSK3β, in desmin phosphorylation in ischemic HF, leading to accumulation and aggregation of desmin.

In the experimental rat model of HF, we have previously shown a modulation of soluble phosphorylated desmin levels [4]. Here, we found a significant increase of the desmin levels in hearts of patients with end-stage ischemic HF, as previously shown [10, 16, 33, 34] but also a significant increase of insoluble CryAB. It is well known that desmin is subjected to many PTM such as phosphorylation, that severely impacts its ability to polymerize into myofilaments, resulting in desmin filament depolymerization and even desmin aggregation [10, 14, 23]. Desmin intermediate filament destructuration, supported by TEM showing disruption of sarcomeres, could alter the structural and mechanical integrity of the contractile apparatus of cardiomyocytes resulting in the decrease of the cardiac contractile capacity. In this context, we postulated that the phosphorylated forms of desmin could be a key step for downstream maladaptive desmin assembly and thus, for adverse LV remodeling post-MI. We then aimed to identify the molecular mechanisms responsible for the modulation of phosphorylated desmin levels during HF and to understand the defective processes leading to the accumulation of desmin aggregates in failing hearts of rats and patients.

An in silico screening of kinases along with an assessment of their activity and/or expression in an experimental rat model of ischemic HF allowed us to highlight the implication of PKCζ and GSK3α/β in the modulation of desmin phosphorylation, as suggested by previous publications [10, 35, 36]. Both were activated when the profile of phosphorylated desmin was modulated in the LV of HF-rats. The role of PKCζ in the heart is poorly understood; however, PKCζ appears to be involved in the hypertrophic response of cardiomyocytes through the regulation of atrial natriuretic factor re-expression [37]. The role of GSK3 α/β in the heart is, however, much better understood. Indeed, GSK3β has been shown to be involved in the anti-hypertrophic processes in the heart [38]. Its inactivation via the phosphorylation of its serine 9 in response to hypertrophic stimuli promotes cardiac hypertrophy and HF development [39]. Using primary cultures of rat neonate cardiomyocytes, we confirmed that PKCζ is able to phosphorylate desmin, in agreement with previous publications [36].

In response to a severe and prolonged stress such as MI, protein homeostasis also called “proteostasis” is disturbed, leading to an accumulation of protein aggregates with consequent proteotoxicity [40]. The heart is particularly susceptible to proteotoxicity because sustained and severe proteotoxic stress leads to cell death, due to its limited self-renewal capacity for maintaining cardiomyocyte homeostasis and integrity [18]. Autophagy mechanisms and the UPS are the major and complementary proteolytic pathways required to eliminate misfolded proteins and defective organelles in most of cells including cardiomyocytes [41]. Alterations of autophagy and/or UPS functions are often associated with the accumulation of proteotoxic species in the heart [42, 43]. We have therefore explored the dynamics of autophagy (macroautophagy and CMA) and UPS in the rat experimental HF model in order to decipher whether altered autophagy occurs during HF development, leading to desmin aggrephagy.

As expected, UPS was not modulated in LV of HF-rats and human failing hearts as previously shown in human failing hearts [32]. We observed a defect in macroautophagy in LV of HF-rats as confirmed by the absence of double-membrane vesicles analyzed by TEM and as previously shown in human failing hearts [32]. Recently, inability of complete autophagy leading to disorganization of cardiomyocyte desmin/mitochondrial network has been described in an experimental rat model of pressure overload-induced HF [25]. More interestingly, we demonstrated for the first time an induction of CMA during LVR post-MI, which may be involved in desmin clearance via its 4 KFERQ-like CMA motifs [27]. Our data showed a switch between macroautophagy and CMA in rats at 2 months post-MI as shown during aging process [44].

We used several modulators of UPS, macroautophagy, and CMA in cardiomyocytes in order to determine the autophagic processes able to eliminate toxic desmin aggregates in order to understand the physiopathological mechanisms during HF development. We observed that activation of macroautophagy or CMA is sufficient for clearance of soluble desmin without any effect on the insoluble form and in its chaperone, CryAB. To strengthen our data, we verified the increased targeting of desmin in purified lysosomes under CMA activation and the presence of a cleaved form of desmin which may result from a cleavage carried out by cathepsin B [31].

Interestingly, ubiquitination of proteins associated with CMA activation are required to prevent desmin aggregates with clearance of both forms of desmin (soluble and insoluble) associated with a decrease of CryAB. This effect is independent of macroautophagy activation as the same effect was observed with combination of UPS and macroautophagy inhibition. Our data are in agreement with publications showing cleaved form of desmin in cytoplasmic aggregates [34, 45]. Our data suggest that despite the ability of CMA to mediate clearance of desmin in vitro in cardiomyocytes, in the in vivo model of ischemic HF, CMA is unable to clear phosphorylated desmin which forms aggregates, mainly detected in the insoluble protein fraction, suggesting the difficulty of the KFERQ motifs to be accessible to Hsc70 [44] but also the requirement of ubiquitination of proteins.

We confirmed in human failing hearts a defect in autophagic processes leading to the presence of insoluble desmin aggregates. Only a trend increase of LAMP2a levels was observed but no modulation of Hsc70 and Hsp90 levels, in accordance with data showing that Hsc70 become limiting when LAMP2a is in excess [44]. Interestingly, significantly increased levels of insoluble CryAB, were found in failing human hearts, and it has been shown that desmin and its chaperone can interplay in the maintenance of mitochondrial homeostasis and cardiomyocyte survival [46, 47]. Our data suggest that CryAB as co-chaperone associated with Hsc70, interacts with hyperphosphorylated desmin aggregates and mediates their degradation through a process named chaperone-assisted selective autophagy (CASA) [48]. Degradation of damaged components through CASA has been described to be activated when CMA is malfunctioning like in LAMP2 k/o mice leading to accumulation of autophagic vacuoles in cardiac muscles [48]. This hypothesis is strengthened by recent publication showing that desmin generate amyloid-like oligomers in a proteotoxic mice model [12]. In the failing human hearts, representing end-stage of the disease, CASA through Hsc70 and CryAB may act as compensatory and adaptative autophagic mechanism for hyperphosphorylated desmin aggregates [49].

### Conclusion and perspectives

HF remains a leading cause of mortality and morbidity and during its development there is an accumulation of toxic aggregates in the heart due to a defective autophagy. Our data demonstrate a novel signaling pathway involved in desmin phosphorylation in ischemic HF. They suggest for the first time that desmin is new substrate of CMA and that CMA is able to clear monomeric phosphorylated soluble desmin. Desmin phosphorylation is complex, being an event that participates in the depolymerization of desmin intermediate filaments and in improper desmin assembly into aberrant aggregates [12]. In pathophysiological conditions, CMA alone without the cooperation of other proteolytic systems, in particular ubiquitination and CASA, is not sufficient for intracellular clearance of phosphorylated desmin but requires the contribution of its chaperone protein, CryaB. Transient soluble phosphorylated desmin may gradually accumulate as insoluble amyloid-like oligomers after MI and thus participate to contractile dysfunction leading to HF. We could speculate that improving autophagy processes earlier after MI by early CMA activation associated with the contribution of CryAB to stimulate desmin clearance might be a therapeutic approach to fight against desmin aggregates in order to maintain cardiomyocyte survival.

## Materials and methods

### Pathway network analysis

Integrated pathway enrichment analysis was performed by using the knowledge-based canonical pathways and endogenous metabolic pathways MetaCore pathway analysis software (GeneGo). The differentially expressed [50] or phosphorylated [3] proteins were mapped into biological networks by using a manually curated proprietary database (MetaCore, GeneGo, St. Joseph, MI, USA), a pathway analysis tool. UniProt protein accession numbers provided from the UniProtKB database (http://www.uniprot.org) were uploaded into the database. For network analysis, expand by one interaction algorithm was used with phosphorylation/dephosphorylation filters. We also used advanced options by selecting both directions, disconnected root nodes, and discarding low trust interactions, functional interactions, and binding interactions.

### Experimental rat model of ischemic heart failure

All animal experiments were performed according to the Guide for the Care and Use of Laboratory Animals published by the US National Institutes of Health (NIH publication NO1-OD-4-2-139, revised in 2011). Randomized animals were used and experimental protocols performed under the supervision of a person authorized to perform experiments on live animals (F. Pinet: 59-350126). Approval was granted by the institutional ethics review board (“Comité d’Ethique pour l’Experimentation Animale Nord Pas-de-Calais” N°242011, January 2012). In brief, rats were anesthetized (xylazine 5 mg/kg intraperitoneally) as previously described [51]. MI was induced in 10-week-old male Wistar rats (Janvier, Le Genest St Isle, France) by ligation of the left anterior descending coronary artery according to the method previously described [1, 51]. Hemodynamic and echocardiographic measurements were performed 2 months after surgery as previously described [50] and are summarized in Table S1. After sacrifice of rats by an overdose of pentobarbital, their hearts were excised and dissected, the right and left ventricles were separated and the infarcted area was removed. Tissues were kept at −80 °C until analysis.

### Transmission electronic microscopy

Rat LVs were immediately fixed in 4% paraformaldehyde and 1% glutaraldehyde in 0.1 mol/L phosphate buffer for 3 h. LV were then rinsed with PBS 1X, cut into 1-mm thickness pieces, and incubated for 1 h in 0.1 mol/L sodium cacodylate containing 1% glutaraldehyde. The samples were post-fixed in the dark for 1 h in 1% osmium tetroxide/1.5% potassium hexacyanoferrate. Several washes with deionized water were performed before incubation of samples in the dark for 1 h in 4% uranyl acetate. The tissues were then rinsed in deionized water and dehydrated through increasing concentrations of ethanol (50%, 70%, 95%, 100%, and 100%, 10 min each) before overnight incubation in a 1:1 mixture of ethanol/epoxy resin. The tissue pieces were embedded in fresh 100% epoxy resin containing 1.7% dimethylaminoethanol for 3 days before epoxy resin polymerization for 24 h at 60 °C. The blocks were trimmed and 80-nm sections cut using a diamond knife, were collected on 100 mesh copper grids. Sections were observed using a Hitachi H7500 transmission electron microscope at 80 kV and photographed using an AMT digital camera.

### Human hearts

Tissues from failing (*n* = 6) and non-failing (*n* = 6) human hearts were obtained respectively from Lille University Hospital (France) and from Catholic University of Leuven (Belgium). Explanted heart tissues were obtained from patients undergoing heart transplantation for end-stage ischemic heart failure and from patients died of non-ischemic causes including suicide (*n* = 1), car crash (*n* = 1), polytrauma (*n* = 1), and trauma (*n* = 3). Samples were quick-frozen and stored at −80 °C. All materials from patients and controls were recovered as surgical waste with informed consent of the donors and with approval of the local ethical boards (“Centre Hospitalier et Universitaire de Lille”, Lille, France and Pole of Pharmacology and Therapeutics of “Université Catholique de Louvain” and “Cliniques Universitaires Saint-Luc”, Brussels, Belgium) and according to the Declaration of Helsinki.

### Cell culture

#### Primary cultures of neonatal rat cardiomyocytes

Primary cultures of rat neonatal contractile cardiac myocytes (NCM) were prepared from heart ventricles of 1- or 2-day-old rats as previously described [22]. Briefly, cardiac cells of newborn rats ventricles were dissociated by enzymatic digestion with 0.04% collagenase II (Worthington, Lakewood, NJ, USA) and 0.05% pancreatin (Sigma-Aldrich). Non-NCM cells were removed by 30 min centrifugation at 1600 × *g* in a discontinuous Percoll gradient (bottom 58.5%, top 40.5%, Sigma-Aldrich). NCM were then seeded at a density of 4 × 10^5^ cells per well in six-well plates coated with 0.01% of collagen (Sigma-Aldrich) and cultured in a medium containing DMEM/Medium199 (4:1), 10% horse serum (Life Technologies), 5% fetal bovine serum (FBS) (ATCC), 1% penicillin and streptomycin (10,000 U/mL, Life Technologies) for 7 days at 37 °C under 5% CO_2_ atmosphere.

To inhibit protein kinase C (PKC) ζ, NCM were serum starved for 1 h and then incubated with 10 µmol/L of the myristoylated PKCζ pseudosubstrate (^114^-IYRRGARRWRKL-^125^, myrPS, Enzo Life Sciences) for 1 h.

To induce macroautophagy, NCM were treated by HBSS (Hank’s Balanced Salt Solution) (Sigma-Aldrich, 55037C) for 2 h and compared to cells cultured in 10% FBS medium. To inhibit the autophagosome-lysosome fusion, NCM were pre-treated with bafilomycin (Baf, 50 nmol/L) for 2 h before co-treatment of the cells with HBSS and Baf for 2 h.

To induce CMA, two pharmacological treatments were used: 2 and 5 µmol/L of geldanamycin (GA, Sigma-Aldrich) for 17 h [30] and pretreatment for 1 h with 10 mmol/L of a macroautophagy inhibitor (3-methyladenine, 3MA, Sigma-Aldrich), and then co-treatment for 18 h with 10 mmol/L 3MA and 10 µmol/L of a proteasome inhibitor (MG132, Sigma-Aldrich).

#### H9c2 cells

Cells were cultured at 37 °C in the presence of 5% CO_2_ in DMEM GlutaMax-1 (Life Technologies) supplemented into 10% FBS. Isolation of lysosomes was performed according to the method previously described with minor modifications [52].

Isolation of lysosomes from H9c2 cells was performed according to the method previously described with minor modifications [52]. Briefly, 8 × 10^6^ H9c2 cells were washed twice with PBS and collected in 1 mL of homogenization medium (HM; 0.25 mol/L sucrose, 1 mmol/L Na_2_EDTA, 10 mmol/L HEPES, pH 7.4) before 10 min centrifugation at 500 × *g*. Cells were then lysed with a Potter-Elvehjem in 1 mL of HM and centrifuged 10 min at 800 × *g*. The supernatant was kept on ice and the pellet was suspended in 500 µL of HM and again centrifuged. The supernatants were pooled and mixed with 120 µL 10% FBS, 670 µL of Percoll solution (90% Percoll, 0.25 mol/L sucrose, 1 mmol/L Na_2_EDTA, 10 mmol/L HEPES, pH 7.4) and HM to a final volume of 3 mL before 45 min ultracentrifugation at 36,000 × *g* and 4 °C. The gradient dense-end-first was collected and supplemented with Igepal CA-630 at a final concentration of 0.5% before pelleting the Percoll by ultracentrifugation for 2 h at 100,000 × *g* and 4 °C. The supernatant was then diluted in 8 volumes of cold acetone and incubated overnight at −20 °C to precipitate the proteins. After a 10 min centrifugation at 15,300 × *g* and 4 °C, the lysosomal proteins were suspended in Laemmli buffer and analyzed by western blot.

### Immunofluorescence

Frozen rat LV tissues and human heart tissues were included in Richard-Allan Scientific™ Neg-50^TM^ (ThermoFisher Scientific Inc). The LV tissue sections (10 µm thickness) and cultured NCMs were fixed for 20 min with 4% paraformaldehyde/PBS and then permeabilized with 0.1% Triton/PBS for 20 min before 30 min incubation in 1% FBS/PBS. After overnight incubation with primary antibodies (see below the detailed list) in 1% FBS/PBS at 4 °C, the samples were incubated for 30 min at room temperature (RT) with a fluorescent conjugated secondary antibody (Alexa Fluor 488 or 555 coupled anti-mouse or anti-rabbit secondary antibodies) diluted 1/500 in 1% FBS/PBS. The LV tissue sections were rinsed with PBS before 10 min nuclei staining with Hoechst 33258 (10 µg/mL) (ThermoFisher Scientific Inc). After washing with PBS, coverslips were mounted with glycerol/PBS 10X (90/10, v/v). Staining was visualized with the ×40 and ×63 objectives of LSM710 confocal microscope followed by Zen image acquisition and software analysis (Zeiss). Images were acquired with a resolution of at least 1024 × 1024 and analyzed with ImageJ software. At least 10 fields are photographed, corresponding to ~100 (×40 objective) −200 (×63 objective) cells depending on the magnification and the type of cells. Photographs shown are representative of the staining observed. Colocalization was quantified by Pearson’s coefficient, describing the correlation between the intensities of 2 proteins with the JACoP plugins on ImageJ software.

We performed proximity ligation assay (PLA) following manufacturer’s instructions (Olink Bioscience, Uppsala, Sweden). Briefly, after primary antibody incubation as described before, samples were incubated 1 h at 37 °C with secondary antibody coupled to oligonucleotidic probes (Duolink® In Situ PLA® Probe anti-mouse PLUS et anti-Rabbit MINUS, Sigma-Aldrich), diluted at 1/5 in BSA 1%. After two washes with PBS 1X, samples were incubated for 30 min at 37 °C with ligase (1/40 in ligation solution). After two washes with PBS 1X, samples were incubated for 1 h 40 min at 37 °C with polymerase (1/80 in amplification solution). After washing with PBS, coverslips were mounted with glycerol/PBS 10X (90/10, v/v). Staining was visualized with the ×40 objectives of LSM710 confocal microscope followed by Zen image acquisition. Images were acquired with a resolution of at least 1024 × 1024 and analyzed with ImageJ software. Number of spots by cells was quantified by a “home-made” plugin write on ImageJ software.

### Proteins extraction

Proteins from human hearts and rat LVs were extracted from frozen tissues (after removing the infarcted area) with Dounce-Potter homogenization into ice-cold RIPA buffer (50 mmol/L Tris pH 7.4, 150 mmol/L NaCl, 1% Igepal CA-630, 50 mmol/L deoxycholate, and 0.1% SDS) containing antiproteases (complete EDTA-free, Roche Diagnostics), serine/threonine and tyrosine-protein phosphatase inhibitors (Phosphatase inhibitor Cocktail 2 and 3, Sigma-Aldrich) and 1 mM Na_3_VO_4_. After 1 h incubation at 4 °C, the homogenate was centrifuged at 15,300 × *g* for 15 min at 4 °C and the supernatant containing soluble proteins was collected. The pellet containing insoluble proteins was solubilized in urea-thiourea buffer (5 mol/L urea, 2 mol/L thiourea, 50 mmol/L DTT, 0.1% SDS in PBS pH 7.4) and sonicated three times for 6 s at 20 Hz (Vibra Cell, Bioblock Scientific 72442) before 15 min centrifugation at 15,300 × *g* and 4 °C. The supernatant containing insoluble proteins was collected and samples were stored at −80 °C. After pharmacological treatments, cells were rinsed twice with PBS before being mechanically scraped from the plate in 50 µL of ice-cold RIPA buffer. Soluble and insoluble proteins were extracted as described above. Protein concentrations for all samples were determined with a Bradford-based method protein assay (Biorad, Marnes-la-Coquette, France).

### Immunoprecipitation, western blot, and Phos-tag gels

#### Immunoprecipitation

Immunoprecipitation was performed with 50 μg of NCM proteins or LV proteins pre-cleared by incubation with protein A/G magnetic beads (88802, Pierce) for 1 h at 4 °C with gentle shaking. The pre-cleared proteins were then mixed with 5 μL of anti-desmin antibody diluted in 100 μL of RIPA 1X buffer as previously described [4]. After overnight incubation at 4 °C on a rotating device, immune complexes were precipitated at 4 °C for only 2 h on a rotating device with 35 μL of protein A/G magnetic beads washed three times in RIPA 1X buffer (Pierce™ Protein A/G Magnetic Beads Thermo Scientific). The supernatant (Sur) was recuperated and the immunoprecipitated (IP) complexes were then washed four times with 750 μL of RIPA 1X buffer before denaturation of both in Laemmli buffer at 70 °C for 10 min and western blot analysis.

#### Western blot

Soluble (5–50 μg) and insoluble proteins (1–20 μg) were analyzed on SDS-PAGE gels (8, 10, 12, or 15%, depending on the protein analyzed). Proteins were transferred to nitrocellulose membranes and blocked for 1 h in Tris-buffered saline with 0.1% Tween-20 (TBS-T) containing 5% skim milk or FBS with constant shaking. Membranes were then incubated with primary antibodies (see Table S2) diluted in TBS-T with 5% skim milk or FBS overnight at 4 °C with constant shaking. The blots were then washed with TBS-T and incubated at RT with horseradish peroxidase-labeled secondary antibodies diluted in 5% skim milk or FBS/TBS-T for 1 h. Following the secondary incubation, the membranes were washed with TBS-T before blots imaging. Equal protein loading was confirmed using GAPDH, α-tubulin, or sarcomeric actin immunoblotting.

#### Phos-tag gels

Soluble and insoluble proteins were analyzed on 10% gels containing 40 µmol/L of Phos-tag (Wako, Osaka, Japan) and 100 µmol/L of Mn^2+^ for detection of the phosphorylated forms of desmin as previously described [53]. At equivalent phosphorylation levels, the position of the phosphate group can also influence the apparent molecular weight of a protein in a Phos-tag gel.

#### Blots imaging

The Chemidoc XRS+ camera (Biorad) and the Image Lab software were used for blots imaging and densitometry analysis. Membranes were incubated for 5 min with Clarity Western ECL Substrate (Biorad) before imaging. The signal was quantified from the image obtained just before saturation. The band corresponding to the protein of interest was framed within a defined area to express the signal intensity depending on the area. This value was related to the intensity value of the reference protein (GAPDH, α-tubulin, or sarcomeric actin). The values were expressed in arbitrary units (A.U.).

### mRNA isolation and quantitative reverse transcription PCR

Total RNA isolation was prepared from LV tissues using TRI Reagent® (Sigma-Aldrich). Tissues were homogenized with an Ultra-Turrax® at 4 °C and then incubated for 5 min at RT. After 10 min centrifugation at 12,300 × *g* the supernatant containing RNA was collected. One part of chloroform was added for 5 parts of TRI Reagent® containing RNA. Samples were shaked and centrifuged for 12 min at 12,000 × *g*. The supernatant was collected and one volume of isopropanol was added for overnight incubation at −20 °C. After 30 min centrifugation at 13,000 × *g* the pellet was washed with ethanol 70% and centrifuged for 10 min at 12,000 × *g*. The pellet was air dried in ice before RNAse-free water solubilization. RNA samples were heated for 5 min at 85 °C and then stored at −80 °C. Sample RNA quality was checked with a bioanalyzer (Agilent 2100 Bioanalyzer, Agilent Technologies) to determine the RNA integrity number. Total RNA (500 ng) was subjected for first stand cDNA synthesis with the miScript II RT kit (Qiagen) according to the manufacture’s recommendations. After cDNA synthesis, the ASB2β expression was evaluated by quantitative PCR with the miSYBR Green PCR kit (Qiagen) on an Mx3000P Q-PCR System (Agilent Technologies). Hypoxanthine-guanine phosphoribosyltransferase (HPRT) was used as an internal control. The relative ASB2β mRNA expression was quantified using the method 2−ΔΔCt, where −ΔΔCt = ΔCt (HF sample) − ΔCt (Sham sample), and ΔCt = Ct (ASB2β) − Ct (HPRT). PCR primers were ATCTCTTTGTTGCCTAGACC (forward) - CAGAAGAGTGACTCAGCAG (reverse) for ASB2β [8] and ATGGGAGGCCATCACATTGT (forward) and ATGTAATCCAGCAGGTCAGCAA (reverse) for HPRT.

### Statistical analysis

Data expressed as mean ± SEM were analyzed with GraphPad Prism version 6.01 (GraphPad Software, San Diego, CA) and comparisons were performed by Mann–Whitney (two-tailed), one- or two-way analysis of variance (ANOVA) with Tukey’s post hoc test, as appropriate. Results were considered statistically significant if the *P* < 0.05.

## Supplementary information

Figure S1

Figure S2

Figure S3

Figure S4

Figure S5

Figure S6

Figure S7

Supplemental informations
