# Supplementary figures and images for: Desmin aggrephagy in rat and human ischemic heart failure through PKCζ and GSK3β as upstream signaling pathways
Source: Cell Death Discov. 2021 Jun 26;7:153. doi: 10.1038/s41420-021-00549-2 (PMC8257599; doi:10.1038/s41420-021-00549-2)

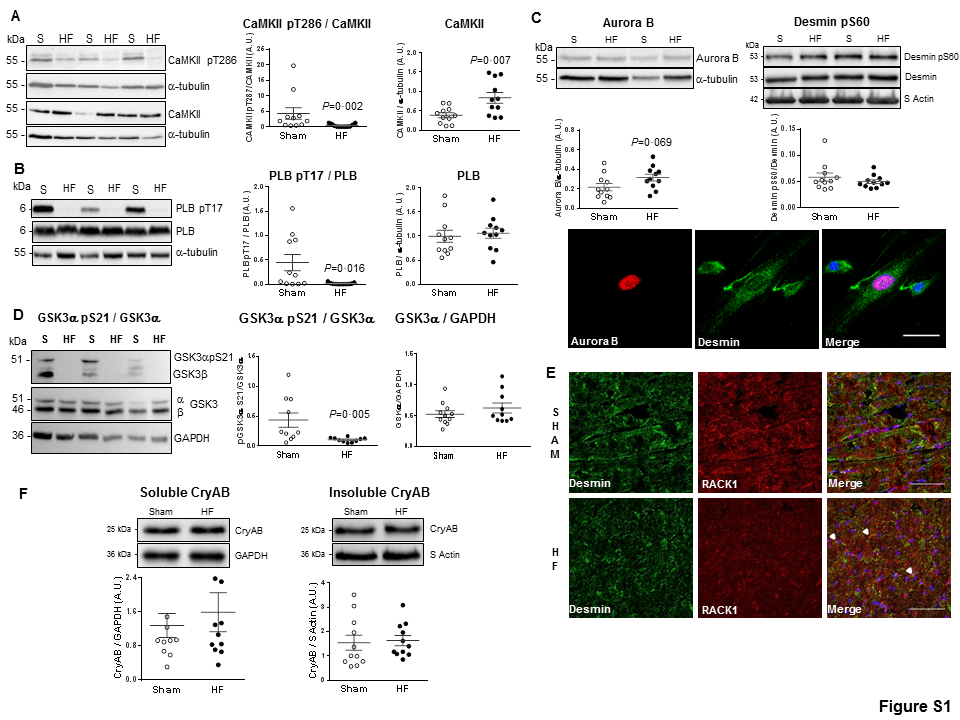

Supplement: Supplementary file 1 — Figure S1 [file 41420_2021_549_MOESM1_ESM.png]

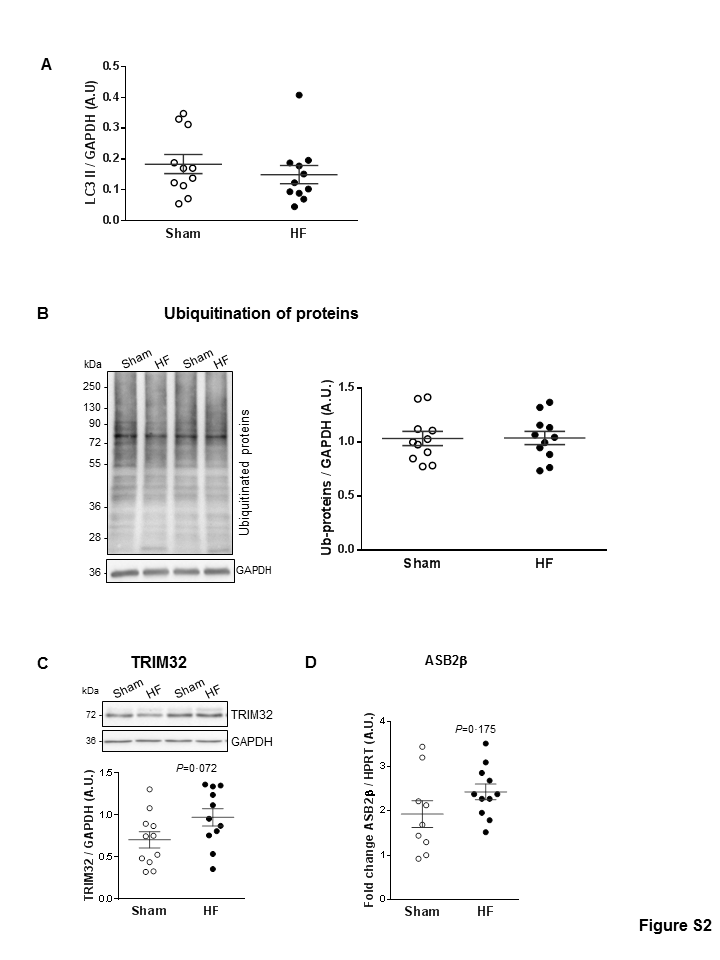

Supplement: Supplementary file 2 — Figure S2 [file 41420_2021_549_MOESM2_ESM.png]

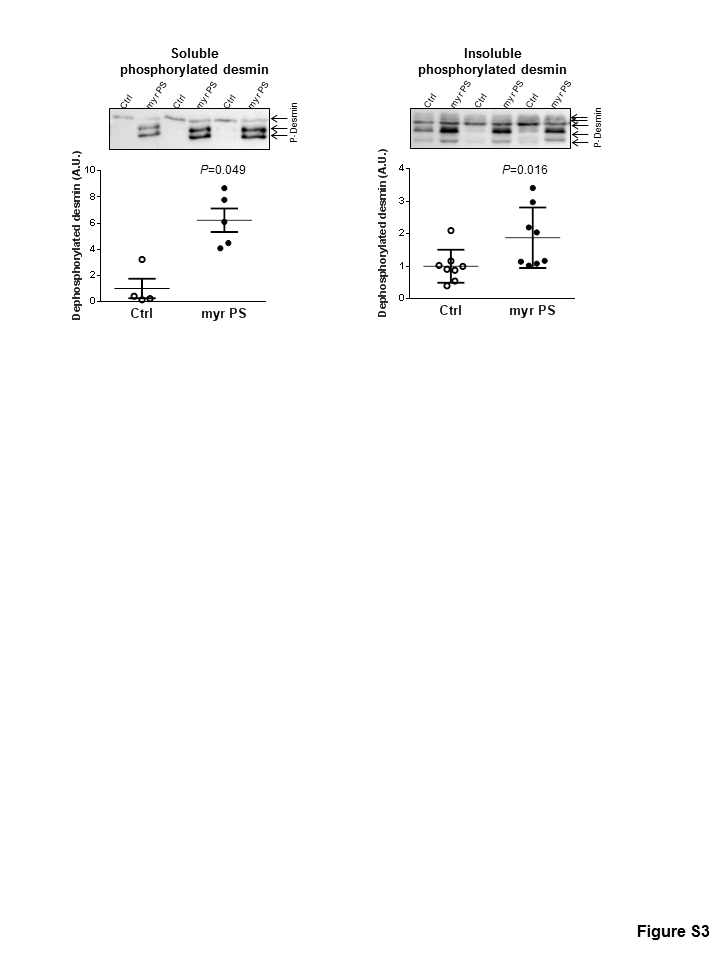

Supplement: Supplementary file 3 — Figure S3 [file 41420_2021_549_MOESM3_ESM.png]

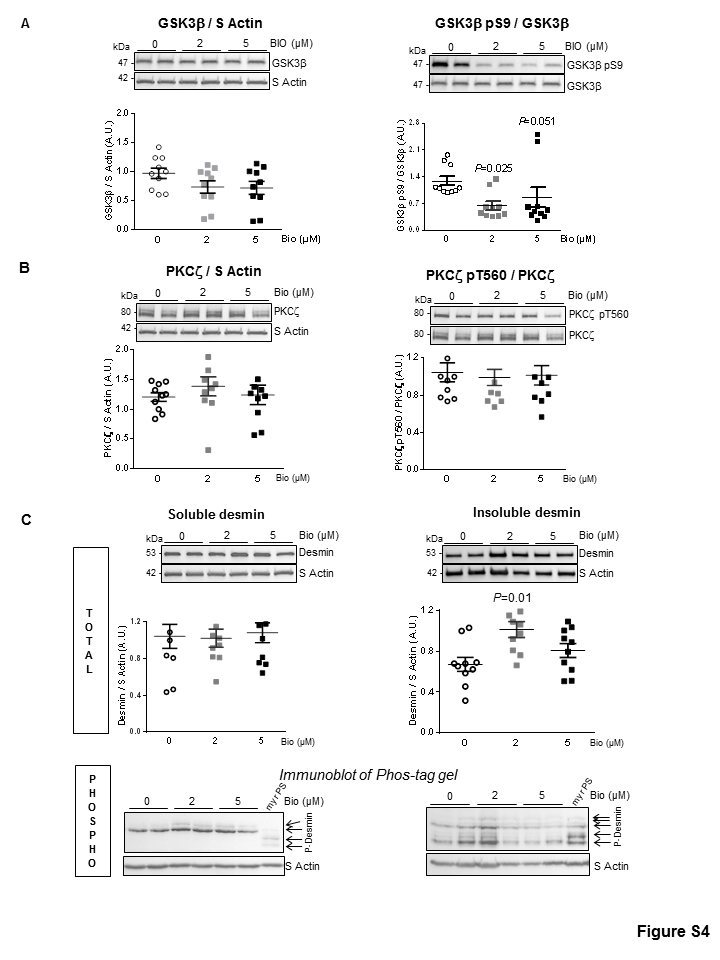

Supplement: Supplementary file 4 — Figure S4 [file 41420_2021_549_MOESM4_ESM.png]

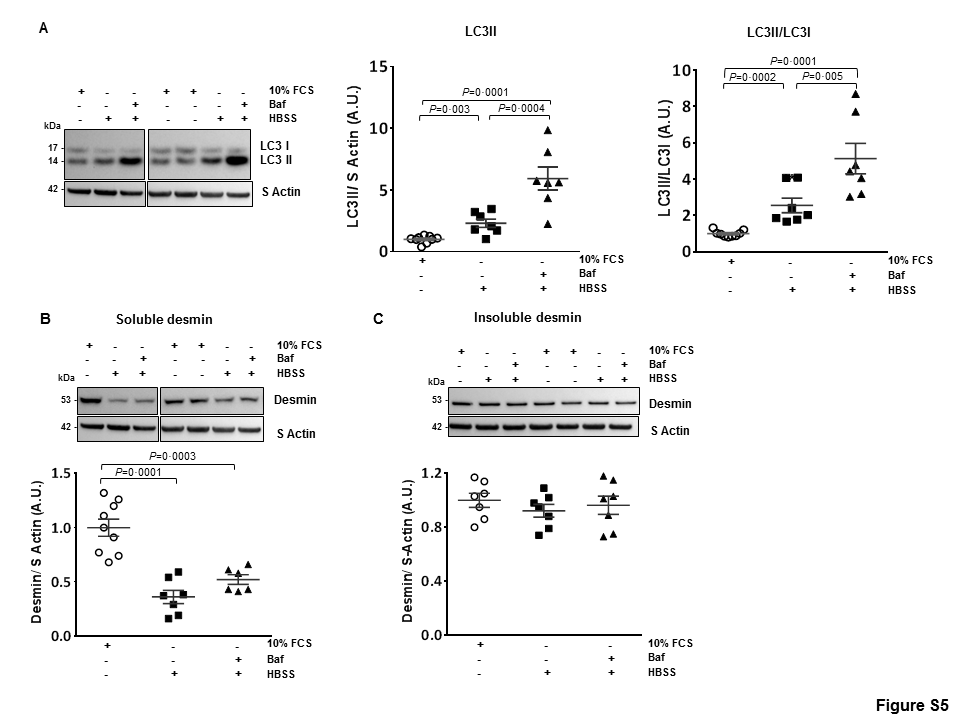

Supplement: Supplementary file 5 — Figure S5 [file 41420_2021_549_MOESM5_ESM.png]

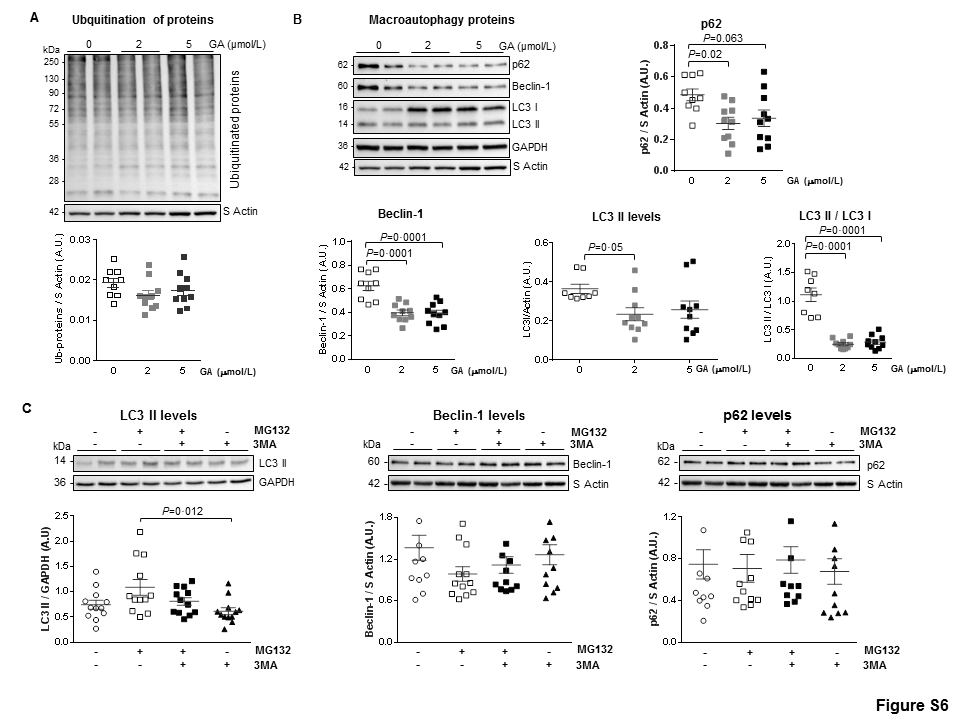

Supplement: Supplementary file 6 — Figure S6 [file 41420_2021_549_MOESM6_ESM.png]

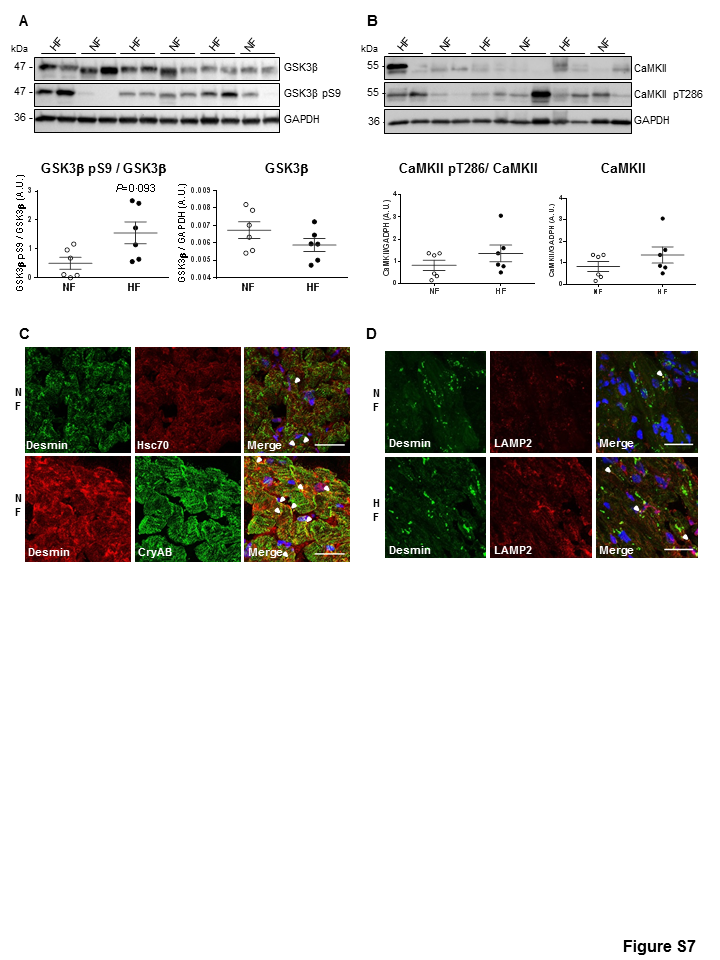

Supplement: Supplementary file 7 — Figure S7 [file 41420_2021_549_MOESM7_ESM.png]
