# Supplementary material for: Desmin aggrephagy in rat and human ischemic heart failure through PKCζ and GSK3β as upstream signaling pathways
Source: Cell Death Discov. 2021 Jun 26;7:153. doi: 10.1038/s41420-021-00549-2 (PMC8257599; doi:10.1038/s41420-021-00549-2)
Supplement: Supplementary file 8 — Supplemental informations [file 41420_2021_549_MOESM8_ESM.docx]

**Supplementary data**

**Desmin aggrephagy in rat and human ischemic heart failure through** **PKC ζ and GSK3β as upstream signaling pathways**

**Running title: Desmin aggrephagy in heart failure**

Marion Bouvet^1^, Emilie Dubois-Deruy^1#^, Annie Turkieh^1#^, Victoriane Peugnet^1^, Paul Mulder^2^, Arthur Dechaumes^1^, Maggy Chwastyniak^1^, Olivia Beseme^1^, Philippe Amouyel^1^, Vincent Richard^2^, Nicolas Lamblin^1^, Florence Pinet^1*^

**Table S1. Histomorphometric and echocardiographic parameters of rats at 2 months post-MI**

| **Parameters** | **Sham**  **(n=11)** | **2 months post-MI**  **(n=11)** |
| --- | --- | --- |
| BW (g) | 453 ± 7 | 444 ± 14 |
| HW (g) | 1·38 ± 0·03 | 1·76 ± 0·11*** |
| LVW (g) | 1·02 ± 0·02 | 1·29 ± 0·07** |
| (HW/ BW)*10^3^ | 3 ± 0·1 | 3·6 ± 0·4** |
| (LVW/BW)*10^3^ | 2·25 ± 0·1 | 2·65 ± 0·3** |
| LVEDD (mm) | 6·7 ± 0·2 | 10·1 ± 0.4*** |
| LVESD (mm) | 3·6 ± 0·3 | 8·4 ± 0·6*** |
| FS (%) | 47·1 ± 2 | 18·5 ± 4*** |
| HR (bpm) | 434 ± 20 | 421 ± 13 |
| SV (ml) | 0·38 ± 0·2 | 0·33 ± 0·03 |
| CO (ml/min) | 167 ± 12 | 137 ± 11 |

MI: myocardial infarction, BW: body weight, HW: heart weight, LVW: left ventricular weight, LVEDD: left ventricular end-diastolic diameter, LVESD: left ventricular end-systolic diameter, FS: fractional shortening, HR: Heart rate, SV: stroke volume, CO: cardiac output. Statistical significance was determined by Wilcoxon-Mann Whitney test. ***P*<0.01, ****P*<0.001 *vs* sham rats.

**Table S2. List of antibodies**

| **Protein** | | **Sample*** | **Reference**  **Company** | **Dilution** | |
| --- | --- | --- | --- | --- | --- |
|  |  |  |  | **Western blot** | **Immunofluorescence** |
| Aurora B | Heart | | 611082  BD Transduction Laboratories | 1/1000 | 1/50 |
| Beclin-1 | Heart | | #3738  Cell Signalling | 1/1000 |  |
| CaMKII | Heart | | ab22609  Abcam | 1/1000 (Rat)  1/300 (Human) |  |
| CaMKII pT286 | Heart (rat) | | PA5-17755  Thermo Scientific | 1/1000 |  |
|  | Heart (human) | | ab32678  Abcam | 1/500 |  |
| CryAB | Heart | | ADI-SPA-223-F  Enzo Life Sciences | 1/15000 | 1/200 |
|  | NCM | |  | 1/20000 |  |
| Desmin | Heart | | ab32362  Abcam | 1/1000 | 1/200 |
|  | NCM | |  | 1/5000 | 1/100 |
|  | Heart | | D1033  Sigma |  | 1/100 |
|  | NCM | |  |  |  |
| Desmin pS60 | Heart | | Ab111382  Abcam | 1/2000 |  |
| GAPDH | Heart/NCM | | sc-36562  Santa Cruz | 1/5000 |  |
| GSK3α/β | Heart | | # 5676  Cell signalling | 1/2000 |  |
| GSK3α pS21 | Heart | | #9316  Cell signalling | 1/1000 |  |
| GSK3β | Heart | | #9315  Cell Signalling | 1/10000 |  |
|  | NCM | |  | 1/5000 |  |
| GSK3β pS9 | Heart/NCM | | #9336  Cell Signalling | 1/2000 |  |
| Hsc70 | Heart/NCM | | ab2788  Abcam | 1/20000 | 1/200 |
| Hsp90 | Heart | | ab13492  Abcam | 1/500 |  |
|  | NCM | |  | 1/1000 |  |
| LAMP1 | NCM | | ADI-VAM-EN001  Enzo Life Sciences | 1/1000 | 1/50 |
| LAMP2a | Heart | | 51-2200  Invitrogen | 1/2000 | 1/50 |
|  | NCM | |  | 1/1000 |  |
| LC3B | Heart/NCM | | #2775  Cell Signalling | 1/1000 |  |
| p62 | Heart/NCM | | 610498  BD Transduction Laboratories | 1/1000 |  |
| Phospholamban | Heart | | sc-21923  Santa Cruz | 1/500 |  |
| Phospholamban pT17 | Heart | | sc-17024-R  Santa Cruz | 1/2000 |  |
| PKCζ C-20 | NCM/H9c2 | | sc-216  Santa Cruz | 1/1000 | 1/100 |
| PKCζ N-17 | Heart | | sc-7262  Santa Cruz | 1/2000 |  |
| PKCζ pT560 | Heart/NCM /H9c2 | | ab62375  Abcam | 1/1000 | 1/100 |
| RACK1 | Heart | | sc-17754  Santa Cruz | 1/1000 | 1/100 |
| Sarcomeric actin | Heart /NCM | | MO874  Dako | 1/2000 |  |
| TRIM32 | Heart | | LS-B1105/8586  LifeSpan Biosciences | 1/5000 |  |
| Mono- and poly-ubiquitin | Heart/NCM | | BML-PW8810  Enzo Life Sciences | 1/2000 |  |
| α-tubulin | Heart | | sc-5286  Santa Cruz | 1/10000 |  |

*heart indicates rat species, otherwise it is indicated.

**Table S3. Correlation between the intensity of desmin with PKCζ, LAMP2a, Hsc70 and CyyAB intensities calculated from double immunofluorescence studies.**

| **Samples** | **Desmin** | | | |
| --- | --- | --- | --- | --- |
|  | **PKCζ** | **LAMP2a** | **Hsc70** | **RACK1/CryAB** |
| **Rat LV**  sham  HF | **0.21** ± 0.02 (4)  0.17 ± 0.02 (4) | **0.22** ± 0.01 (6)  **0.20** ± 0.03 (2) | 0.09 ± 0.02 (3)  0.12 ± 0.04 (2) | **RACK1**  0.18 ± 0.01 (3)  0.15 ± 0.01 (3) |
| **NCM**  Ctrl  GA  Ctrl  MG132+3MA | **0.25** ± 0.06 (3)  **0.47** ± 0.02* (2) | 0.16 ± 0.04 (3)  **0.23** ± 0.03 (8) | ***0.41*** ± 0.07 (6)  ***0.40*** ± 0.04 (13) |  |
|  |  | **0.32** ± 0.02 (3)  **0.26** ± 0.04 (3) | **0.23** ± 0.02 (3)  **0.36** ± 0.02 (2) |  |
| **Human heart**  non-failing  failing | **0.33** ± 0.06 (8)  ***0.63*** ± 0.03^#^ (9) | **0.37** ± 0.06 (8)  **0.24** ± 0.03 (15) | **0.35** ± 0.01 (5)  **0.33** ± 0.04 (7) | **CRYAB**  **0.22** ± 0.17 (5)  **0.37** ± 0.15 (13) |

A correlation (Pearson coefficient) between the intensities quantifies the degree of colocalization between two labellings and reflects either a direct molecular interaction or an indirect interaction. A Coefficient Interval (CI) <0.199 is considered Very Weak, a CI between 0.20-0.399 is Weak (bold), a CI between 0.40-0.599 is Medium (bold and italic) and a CI between 0.60-0.799 Strong (bold, italic and underlined). (Number) indicates the number of replicates. **P*<0.05 *vs* CTRL, # *P*<0.001 *vs* failing human heart.

**Legends of supplemental figures**

**Figure S1. Regulation of kinases potentially involved in desmin phosphorylation, identified by bioinformatic analysis. A:** Representative western blots (left panel) and quantiﬁcation of active CaMKII (CaMKII pT286/CaMKII ratio) (middle panel) and CaMKII levels (right panel) in LV of sham- (n=11) and HF-rats 2 months post-MI (n=11). **B:** Representative western blots (left panel) and quantiﬁcation of phosphorylated phospholamban (PLB) on threonine 17 (PLB pT17/PLB ratio) (middle panel) and PLB levels (right panel) in the same samples. **C:** Representative western blots and quantiﬁcation of Aurora B (top left panel) and of phosphorylated desmin at S60 (desmin pS60/desmin ratio) (top right panel) in the same samples. Double immunofluorescence staining for desmin (green) with Aurora B (red) in H9c2 cells (bottom panel). Nuclei are stained in blue. Scale bar represents 40 µm. **D:** Representative western blots (left panel) and quantification of inactive GSK3α (GSK3α pS21/GSK3α ratio) (middle panel) and GSK3β levels (right panel) in the same samples. **E:** Double immunofluorescence staining for desmin (green) with RACK1 (red) in the LV of sham- and HF-rats 2 months post-MI. Arrows indicates the colocalisation of desmin with RACK1. Scale bar represents 60 µm. **F:** Representative western blots and quantiﬁcation of CryaB in soluble (left panel) and insoluble protein fractions (right panel) in the same samples. The loading controls (GAPDH, S Actin and α–tubulin) are indicated on the graphs. Graphs show individual and mean ± SEM values expressed in arbitrary units (A.U.). Significant *P* values are indicated on the graphs.

**Figure S2. Ubiquitin proteasome system (UPS) is involved in desmin clearance. A**: Quantiﬁcation of LC3II levels in LV of sham- (n=11) and HF 2 months post-MI rats (n=11). **B**: Representative western blots and quantiﬁcation of ubiquitinated proteins in the same samples. **C:** Representative western blots and quantiﬁcation of TRIM32 levels in the same samples. **D:** ASB2β mRNA expression was quantified in the same samples by qPCR with Hypoxanthine-guanine phosphoribosyltransferase (HPRT) used as internal control. Graphs show individual and mean ± SEM values expressed in arbitrary units (A.U) except for ASB2β expressed as fold change. Significant *P* values are indicated on the graphs.

**Figure S3.** Phosphorylation profiles (Phos-tag™ gel) of soluble (left panels) and insoluble desmin levels (right panels) were analyzed in control and NCM treated with 10 µmol/L of PKCζ inhibitor, myrPS for 1 hr (n=8). Each band of the desmin immunoblot of Phos-tag™ gels represents a phosphorylated form of desmin, the upper band being the most phosphorylated and the lower band the less or no phosphorylated form of desmin. Graphs show individual and mean ± SEM values expressed in arbitrary units (A.U.). *P* values are indicated on the graphs.

**Figure S4. GSK3β modulates desmin phosphorylation *in vitro*.** (**a**) Representative western blots and quantiﬁcation of GSK3β levels (left panel) and active GSK3β (GSK3β pS9/GSK3β ratio) (right panel) in control and NCM treated with 2 and 5 µM of GSK3β inhibitor, BIO for 24 hrs (n=10). (**b**) Representative western blots and quantiﬁcation of PKCξ levels (left panel) and active PKCξ (PKCξ pT560/PKCξ ratio) (right panel) in the same samples. (**c**) Soluble (left panels) and insoluble desmin levels (right panels) and their phosphorylation profiles (Phos-tag™ gel, bottom panels) were analyzed in response to GSK3β inhibition in the same samples. To compare the phosphorylation profile of desmin following GSK3β inhibition and PKCξ inhibition, a lane with myrPS treated-NCM was added on the right. Graphs show individual and mean ± SEM values expressed in arbitrary units (A.U.). *P* values are indicated on the graphs.

**Figure S5. Soluble desmin is degraded by nutrient starvation *in vitro* in cardiomyocytes**. **A**: Representative western blots (left panel) and quantiﬁcation (right panels) of LC3II levels and LC3 II/LC3 I ratio in NCM cultured on different conditions: 10% FCS (Fetal Calf Serum) (n=7) and Hank’s Balanced Salt Solution (HBSS) with (n=7) or without (n=7) pretreatment with bafilomycin (Baf, 50 nmol/L) for 2 hrs. **B**: Representative western blots and quantiﬁcation of soluble desmin levels in the same samples. **C**: Representative western blots and quantiﬁcation of insoluble desmin levels in the same samples. Graphs show mean ± SEM values expressed in arbitrary units (A.U.). Significant *P* values are indicated on the graphs.

**Figure S6. Impact of CMA activation by GA (A, B) or MG132 and 3MA (C) on UPS and macroautophagy.** **A**: Representative western blots (top panel) and quantiﬁcation (bottom panel) of ubiquitinated proteins levels in control (n=8) and NCM treated with 2 and 5 µmol/L of GA during 17 hrs (n=10). **B**: Representative western blots and quantiﬁcation of macroautophagy markers, p62, beclin-1, LC3II levels and LC3 II/LC3 I ratio in control (n=8) and NCM treated with 2 and 5 µmol/L of GA during 17 hrs (n=10). **C**: Representative western blots and quantification of LC3 II, beclin-1 and p62 levels in control (n=8) and NCM treated either with MG132 (n=12) or 3MA (n=12) for 18 hrs, and NCM pre-treated with 3MA for 1 hr and then co-treated with 3MA and MG132 for 18 hrs (n=12). Graphs show mean ± SEM values expressed in arbitrary units (A.U.). Significant *P* values are indicated on the graphs.

**Figure S7. Desmin presents a scaffold defect and accumulates in the failing human hearts. A:** Representative western blots (top panel) and quantification of inactive GSK3β (GSK3β pS9/GSK3β ratio) (bottom left panel) and GSK3β levels (bottom right panel) levels in the heart of non-failing (NF) (n=6) and failing (HF) patients (n=6). **B:** Representative western blots (top panel) and quantiﬁcation of active CaMKII (CaMKII pT286/CaMKII ratio) (bottom left panel) and CaMKII levels (bottom right panel) in the same samples. The loading control (GAPDH) is indicated on the graphs. Significant *P* values are indicated on the graphs. **C**: Double immunofluorescence staining for desmin (green) with Hsc70 (red) (top panels) and for desmin (red) with CryAB (green) (bottom panels) in frozen myocardial sections from non-failing patients. Scale bar represents 50 µm. Nuclei are stained in blue as shown in the merge staining. Arrows indicate colocalization of desmin with Hsc70 or CryAB. **D**: Double immunofluorescence staining for desmin (green) with LAMP2a (red) in frozen myocardial sections from non-failing (NF) (top panels) and failing (HF) (bottom panels) patients. Nuclei are stained in blue as shown in the merge staining.Arrows indicate colocalization of desmin with LAMP2a. Arrows indicate colocalization of desmin with LAMP2a. Scale bar represents 50 µm.
